# Nano-inspired fluidic interactivity for boiling heat transfer: impact and criteria

**DOI:** 10.1038/srep34348

**Published:** 2016-10-06

**Authors:** Beom Seok Kim, Geehong Choi, Sangwoo Shin, Thomas Gemming, Hyung Hee Cho

**Affiliations:** 1 IFW Dresden, P. O. Box 270116, 01171 Dresden, Germany; 2 Department of Mechanical Engineering, Yonsei University, Seoul 03722, Korea; 3 Department of Mechanical and Aerospace Engineering, Princeton University, Princeton, New Jersey 08544, Unites States

## Abstract

The enhancement of boiling heat transfer, the most powerful energy-transferring technology, will lead to milestones in the development of high-efficiency, next-generation energy systems. Perceiving nano-inspired interface functionalities from their rough morphologies, we demonstrate interface-induced liquid refreshing is essential to improve heat transfer by intrinsically avoiding Leidenfrost phenomenon. High liquid accessibility of hemi-wicking and catalytic nucleation, triggered by the morphological and hydrodynamic peculiarities of nano-inspired interfaces, contribute to the critical heat flux (CHF) and the heat transfer coefficient (HTC). Our experiments show CHF is a function of universal hydrodynamic characteristics involving interfacial liquid accessibility and HTC is improved with a higher probability of smaller nuclei with less superheat. Considering the interface-induced and bulk liquid accessibility at boiling, we discuss functionalizing the interactivity between an interface and a counteracting fluid seeking to create a novel interface, a so-called smart interface, for a breakthrough in boiling and its pragmatic application in energy systems.

Economic growth and technological advancement have relied on the availability of efficient, cost-effective, sustainable energy; indeed, the development of sustainable energy sources continues to be pressing issue for humankind^1^. Various engineering approaches have attempted to enhance the efficiency of existing power generation systems based on fossil fuel and nuclear fission plant; the issue has also triggered research into a challenging nuclear fusion plant as an alternative and ultimate energy source. Taking into account principal rules in thermodynamics, however, higher operating temperature conditions are essential for achieving higher efficiency in the thermodynamic cycle of most gas-turbine systems. Moreover, nuclear fusion requires high-temperature plasmas, resulting in significant thermal loads of over a few megawatts per square meter in a tokamak system. Thus, there is a need for technology breakthroughs for highly efficient thermal energy transfer schemes and subsequent high technology for cooling.

Convection in heat and mass transfer, defined in terms of the energy transfer via the movement of groups or aggregates of molecules within a fluid, is widely used to describe various cooling and thermal energy transfer processes due to its efficiency and applicability. As a pragmatic convection scheme, boiling heat transfer may be a promising solution to technical demands due to its outstanding heat/energy transfer ability^2^. A goal in boiling research is to maximize this ability, which can be specifically indicated by the allowable heat dissipation capacity (*i.e.*, the critical heat flux, CHF) and the heat dissipation efficiency (*i.e.*, the heat transfer coefficient, HTC). Boiling is a thermo-fluidic phenomenon accompanying the heterogeneous phase change of a working fluid on a surface and multiphase flow, involving cascading ebullition and, subsequently, intricate convective behavior^3^,^4^. On these phenomenological grounds, which reach back to the historic discovery of Leidenfrost in 1756^5^, boiling performance is governed primarily by the hydrodynamic balance between vaporization and the counteracting liquid accessibility at a heat-dissipating hot surface. Thus, the major research streams for boiling enhancement have pursued how to manipulate the hydrodynamic characteristics of a working fluid and to functionalize interface-inspired characteristics with respect to *interfacial* hydrodynamic interactivity^6^,^7^. Figure 1 shows schematically the principal factors in the *bulk* and *interface-inspired* hydrodynamic aspects and subsequent boiling characteristic curves. The bulk fluidic accessibility is related to the hydrodynamic balance between vapor and counteracting liquid, and the interface-inspired accessibility is explained with morphology-induced interfacial effects involving static and dynamic wetting^8^.

Novel nano/material technologies for nano-inspired functional interfaces have triggered a possible leap in boiling research, due to their unique characteristics^9^,^10^; these features originate from the intrinsic functionality of extremely rough morphologies and associated fluidic interactivity^11^,^12^,^13^,^14^. Extremely high surface roughness, via nanoscale structures, such as vertically aligned nanowires^10^,^14^,^15^,^16^ and micro/nano-hierarchical structures^13^,^17^, greatly expand the interfacial contact area, allowing greater heat dissipation than macroscale structures do. Additionally, surface roughness accompanies the intensification of hydrophilicity, towards a superhydrophilic regime, which is clearly favorable to wetting or refreshing of the interface by a liquid-phase working fluid.

Although boiling performance has been improved markedly by the use of novel nanomaterial technologies, there are unresolved issues regarding CHF^2^,^12^. What are the direct grounds for the recent breakthroughs in enhancing CHF using nano-inspired surfaces? Can we suggest a plausible and universal CHF model predicting the CHF as a physical function of the design variables of the interfacial surfaces? In particular, previous approaches primarily considered the two factors of surface roughness and static wetting characteristics, which are not independent factors but tied up each other. The factors were unable to provide a plausible physical demonstration revealing peculiar contributions of nanoscale structures to boiling enhancement^10^,^12^,^18^. This raises questions as to whether the principal factors considered so far are indeed independent and whether other as-yet undiscovered factors may explain the phenomenon. In considering this, we bring up an additional peculiarity from nano-inspired functionality: that is, the interfacial hydrodynamics regarding dynamic wetting by morphologically driven hemi-wicking^2^,^15^,^19^,^20^,^21^,^22^. Beyond intuitive concepts regarding surface roughness and static wettability, morphologically driven hemi-wicking, which can be an integrative index of the intuitive features, has proven to be a governing factor determining the interfacial hydrodynamic status^15^,^19^,^20^,^21^,^22^.

In this study, we provide a plausible answer to the question as to whether interfacial hemi-wicking is an appropriate factor describing the peculiar role of nano-inspired surfaces and suggest a prerequisite for a future boiling technique. We confirm that the initiation of hemi-wicking, which must be differentiated from unconditional surface roughening, contributes greatly to the enhancement of CHF by reinforcing interfacial re-wetting^2^,^12^,^23^. Moreover, the causative nano-inspired functionality, which especially accompanies apparent superhydrophilicity, leads to an increase in HTC by catalyzing heterogeneous nucleation, due to the higher probability of smaller nuclei with less wall superheat demanded. For the demonstration of separate role of intrinsic liquid accessibility and morphologically-driven accessibility, we use a surfactant in order to strengthen bulk spreading of fluid on nano-treated surfaces. We demonstrate the independent impacts of *interface-induced* and *bulk* liquid accessibilities on boiling and examine their superpositioning effects for heat dissipation performance and their limitations. *Bulk* fluidic accessibility is controlled by the surface tension *σ* of the working fluid (here, deionized (DI) water) without affecting other bulk properties using a surfactant additive (FS-3100, DuPont). Local heal transfer characteristics are evaluated by a sensor (Fig. 2(a,b)) devised with different type of *in situ* nanostructures of vertically aligned silicon nanowires (SiNWs, Fig. 2(c,d)) and silicon nanopillars (SiNPs, Fig. 2(e–h)). The local temperature measuring sensors are just below the heating area (1 × 0.5 cm^2^) and an indium tin oxide layer is for a thin film heater. Randomly dispersed SiNWs and regular SiNPs are confined by a universal factor of the solid fraction (*φ*) defining morphology, which remains dry when the structures contact a liquid droplet. (See Methods section and Supplementary Information for material preparations, experimental procedure and data reduction).

## Results and Discussion

### CHF

Convective boiling behavior is explained by a fluidic aspect characterizing the phase change of a fluid and sequential multiphase flow^4^,^8^. Figure 3(a) shows typical behavior in terms of the fluidic resistance and hydrodynamic stability between up-flowing vapor columns and counteracting down-flowing liquids towards the boiling surface^6^,^7^,^24^. When stability cannot be maintained, resulting in a lack of refreshing liquids, devastating performance degradation and critical surface burnout will eventually occur due to a vapor blanket on the surface. Here, the surface tension of a bulk fluid is an important parameter affecting liquid accessibility and subsequent convective fluidic behaviors^25^. Thus, it is desirable to establish a quantitative relationship between the depression in liquid surface tension and the increase in the rate of heat transfer in the nucleation boiling regime^26^,^27^. However, nano-inspired interfaces can trigger peculiar *near-surface* hydrodynamic behavior (*i.e.*, hemi-wicking) inducing morphology-induced liquid refreshing against vaporization just above a solid–liquid interface, as described in Fig. 3(b). As it is already classified by previous study^8^, roughness and its impact on dynamic wetting of the hemi-wicking, which are not highlighted by *bulk* so-called *far-field* hydrodynamic mechanisms, can be covered by a *near*-surface mechanism^3^,^12^,^13^,^18^,^28^,^29^.

Figure 4 shows boiling curves that are dependent on the bulk and the interface-induced liquid accessibilities, according to the variation in surface tension of the working fluid and the application of interfacial hemi-wicking, respectively. CHF occurs when the liquid cannot be sufficiently refreshed to maintain a balance against vigorous vaporization at a boiling site. Thus, CHF can be characterized in terms of hydrodynamic performance. The liquid accessibility against vaporization of a working fluid can be intrinsically forced by lowering its surface tension to be favorable for convective boiling^30^,^31^,^32^. However, excessive depression of surface tension over a critical point of additive concentration (*i.e.*, the critical micelle point) can cause convective boiling performance to deteriorate because a further increase in additive materials, lowering *σ*, causes an increase in viscosity, which paradoxically hinders liquid accessibility towards a boiling surface^26^,^27^,^30^,^31^,^32^,^33^. This indicates the presence of an optimal condition for the manipulation of the bulk accessibility, presented in Fig. 4 as parabolic asymptotic distributions of CHFs with variation of concentration on a plain surface. However, hemi-wicking, driven by morphology manipulation, can reinforce liquid refreshing of heterogeneous boiling interfaces; as a factor characterizing the *near-field* hydrodynamic status on solid–liquid interfaces, it directly contributes to the enhancement of CHF. Its impact can be seen in Fig. 4 as a CHF increase, by more than 100%, on a wicking-inducing surface versus non-wicking conditions on a plain surface^12^,^34^.

Considering the two hydrodynamic aspects, we demonstrate a theoretical CHF model based on hydrodynamic instability between upward vapor columns and downward liquid flow (Fig. 3(a)) and interfacial liquid refreshing by hemi-wicking (Fig. 3(b))^12^. Especially for defining the interfacial liquid refreshing via an interface manipulation, we attempt to formulate a model with a universal variable of the solid fraction (*φ*). The stability from *bulk* hydrodynamics should be guaranteed by balancing the down-flowing liquid and up-flowing vapor columns. When the Helmholtz instability of vapor columns leaving the surface distorts and blocks liquid accessibility to a boiling surface, critical heat dissipation will be attained^35^,^36^. As vaporization continues, the surface becomes deprived of liquid refreshing and an insulating vapor layer impedes heat dissipation by covering the surface. Based on Helmholtz stability, the allowed maximum heat dissipation capacity within a stable regime can be defined as the CHF (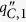)^8^,^37^, and it can be postulated by considering the available gravitational energy to drive liquid into the boiling surface, resulting from the body force and the static wetting characteristics of the surface^29^:


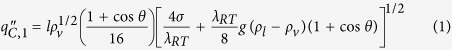


where *l, ρ, θ*, and *g* are the latent heat of vaporization, density, CA on a boiling surface, and gravitational acceleration, respectively. The subscripts *v* and *l* indicate the vapor and liquid phases, respectively. The upward bubble column should be regarded with Helmholtz stability against downward liquid accessibility, and the characteristic length of the bubble column can be substituted by the Rayleigh–Taylor wavelength, *λ*_*RT*_, which defines the critical distance of a stable interface between two fluids of different densities (here, up-flowing vapor and down-flowing liquid, Fig. 3(a))^24^,^38^,^39^. The Rayleigh–Taylor wavelength is given as 2*π*(*σ*/(*g(ρ*_*l*_ − *ρ*_*v*_)))^1*/*2^ for the most critical cases in boiling analysis^24^,^29^,^40^.

Taking into account interface-induced hydrodynamics, we can build a supplementary model, which reflects the interfacial dynamic re-wetting and consequent heterogeneous phase change. The driving force of *interfacial* liquid refreshing is morphologically induced hemi-wicking; thus, we can deduce the refreshing rate of a liquid, *i.e.*, its wickability, *W*^41^,^42^. (See Supplementary Information for characterization of the wickability.) Here, the volumetric refreshing rate of a porous interface filled by hemi-wicking is expressed as *h*·(1 − *φ*)*W*^2^ with the geometrical variables of the height of the employed interfacial structures (*h*) and the solid fraction (*φ*) of the wicking-inducing interface. The total amount of heat dissipation by interfacial re-wetting and the sequential phase-change (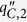) is then specified for the near-field. Then the critical amount of subsequent heat dissipation can be estimated by adopting the prerequisite stable boiling area with the critical characteristic length of *λ*_*RT*_ as follows^12^:


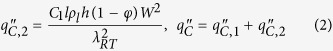


where the shape factor *C*_1_ is a correlating coefficient with regard to *h*, defined as the specific wicking space^12^. Herein, we assume that the hydrodynamic refreshment is uniform on a confined area (*λ*_*RT*_^*2*^) preventing a local gradients that may occur due to the size effects^43^,^44^. This converging approach predicting CHF (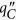) on a blend of the *bulk* and the *interface-induced* hydrodynamics is consistent with experimental results^12^. If we can define surface status with a framed value of the solid fraction, as shown in Fig. 5, it can be a universal one which enables to cover various types of interfacial structures. From the demonstration, we find that morphological design variable *φ* and the sequential capillary momentum of wickability over a manipulated interface are the principal factors determining the CHF. This model shows that hemi-wicking may be an unrevealed clue^2^, providing a plausible physical demonstration of the peculiar contributions of nanoscale structures and a possibility to be a universal model covering the advantages obtainable from various types of nanostructures to boiling enhancement. Interfacial hemi-wicking is generated by the morphology-induced capillary pressure on the roughened surfaces and the consequential stronger interfacial refreshing leads to a greater liquid supply directly to the boiling surface. On this basis, it explains that the amount of critical heat dissipation can be increased by reinforcing capillary flow momentum through a structured forest, as well as by decreasing counteracting fluidic resistance with a lower *φ*^12^,^22^,^41^,^42^. Considering these aspects of boiling surface design, wickability (*W*), subordinate to a universal factor of the solid fraction (*φ*) defining interface characteristics^15^, can be decisive for the plausible extension of CHF according to the suggested model.

### HTC

This peculiarity of nano-inspired functionality can also be highlighted in convective heat dissipation efficiency in boiling. As a heat/mass transfer mechanism, boiling heat dissipation is governed thoroughly by ebullition behavior: nucleation, growth, release, and mixing of the two phase fluids. Liquid accessibility is also a significant factor characterizing ebullition on a heterogeneous liquid–solid interface. Liquid accessibility can be manipulated passively by controlling surface tension, because lowering the surface tension of a bulk fluid leads to a weaker cohesive force between molecules and, consequently, favors accessibility to a boiling surface^7^. This tangible property of a liquid affects embryo evolution into bubble nucleation. For the range of active nucleation sites, vapor nuclei should form with an elevated pressure, higher than that of the surrounding liquid. The pressure difference between the nuclei and surrounding liquid is explained by the Young–Laplace equation (∆*P* = 2*σ*/*r*_*b*_, where *r*_*b*_ is a bubble radius), and the pressure elevation can be obtained by superheat, which is a thermal energy source that results in a temperature gradient in a hypothetical thermal boundary layer on a boiling surface^45^,^46^. In the presence of superheat and the consequent temperature gradient across the layer, the range of active nucleation cavities *r*_*c*_ can be given as a function of the wall superheat by^45^,^46^:


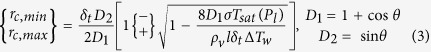


where *δ*_*t*_, *T*_*sat*_, and *∆T*_*w*_ are the thermal boundary layer thickness, saturation temperature of the liquid, and wall superheat, respectively. The two terms *D*_1_ and *D*_2_ reflect the hydrostatic wetting characteristics of the surface. The effective cavity radius *r*_*eff*_ can be defined as the minimum wall superheat (*∆T*_*w, min*_) condition that meets the criterion for nucleation initiation, which can be expressed as *r*_*eff*_ = *r*_*c*_(*σ, θ, ∆T*_*w, min*_). The decrease in *r*_*eff*_ is attributable to the lowering of the liquid surface tension, as shown in Fig. 6, for both surfaces with and without hemi-wicking. On interfaces with and without hemi-wicking, reinforcing direct liquid accessibility by lowering *σ* attenuates the effective cavities. According to this approach, we can further demonstrate its impact on convective heat dissipation and heat transfer stability^11^,^14^. Small nuclei can be dispersed evenly on a roughened heating surface^9^,^10^, and a liquid with lower *σ* can readily access a boiling surface through the secured vacant space between restrained-nuclei; small nuclei can be released more readily and more rapidly, which will consequently lead to enhanced convective heat transfer. It has been demonstrated that convective heat dissipation is more stable and effective with small nuclei than with large bubble formation, because the latter retards the ebullition process from nucleation to release and causes straightforward aggregation into an insulating vapor-film layer^11^,^26^,^27^,^32^,^33^. As shown in the inset of Fig. 6, highly accessible liquids reinforced by passive *σ* lowering via higher surfactant concentration are thus effective in stabilizing temporal wall temperature fluctuations (*i.e., T*_*std*_, the standard deviation of transient wall temperature variations) during nucleation. In the nucleate boiling regime, we confirm that local surface temperature was more stable with attenuated temperature fluctuation by improving liquid accessibility through bulk hydrodynamic aspects even on the nano-treated surfaces.

From lowering the surface tension on an untreated surface without any effects of nano-inspired functionality, a dramatic superheat decrease was observed as a left-shifting of the boiling curves (Fig. 4). However, wall superheat variations were insensitive to nano-inspired surfaces. This different influence of passive liquid feeding from the lowered surface tension of a fluid is more evident if the experimental results are expressed as a plot of HTCs. From Fig. 7, the differential convective boiling characteristics can be found according to the superpositioning of the two strategies: passive (bulk) liquid feeding by lowering surface tension of a fluid and interfacial liquid refreshing by morphologically-induced hemi-wicking. Although the absolute HTCs are improved by lowering *σ* in every case without regard to the presence of hemi-wicking (inset of Fig. 7), the enhancement ratio of HTC_*i*_/HTC_*DI*_ (subscripts *i* and *DI* indicate the *σ*-manipulated cases via a surfactant and DI water, respectively) weakens comparatively on a surface with hemi-wicking than without hemi-wicking by lowering the surface tension. This can be demonstrated based on the degree of *r*_*eff*_ variation (Fig. 6) according to lowering *σ* (from 59.0 to 16.0 mN/m): specifically, a decrease by 4.0 μm on a plain surface without hemi-wicking and by 0.41 μm with hemi-wicking, an order of magnitude difference. The *near-field* interfacial liquid accessibility by hemi-wicking is also feasible for making nucleation effective before bulk liquid feeding, by lowering *σ*^47^. Because the hemi-wicking surface initially confines *r*_*eff*_ to 0.88 μm due to subsequent superhydrophilic characteristics with a CA below 8.9°, it significantly cancels out the direct effects of lowered surface tension on *r*_*eff*_ attenuation.

The weakening of the enhancement ratio, when we directly reinforce *bulk* liquid accessibility, can also be demonstrated intuitively with the wall superheat characteristic and its impact on local Marangoni flow. Nucleated bubbles themselves act as a thermal insulating layer, with a lower thermal conductivity of 0.016 W/m·K, resulting in a considerable wall superheat increase during the nucleate boiling process. However as discussed previously, superhydrophilic characteristics accompanied by hemi-wicking generate predominantly small nuclei ebullition^11^, which constrains the effect of *σ*-dependent nuclei attenuation. Consequently, there is a diminished influence of *σ* on the reduction of wall superheat, which translates into a reduction in temperature deviation between the boiling surface and a saturated bulk fluid. This indicates that Marangoni flow induced by local temperature gradient around the nuclei should be attenuated, when we superpose the two strategies for liquid accessibility. In the case of upward surface and wall-heating conditions, upward liquid flows around the nucleated bubble generated by the thermal Marangoni force (Fig. 8), proportional to (*∂σ/∂T*)·∆*T*_*w*_, which is caused by a surface tension differential along the bubble interface. This Marangoni flow originally prohibits the growth and detachment of nucleated bubbles, resulting in a flow opposite to the thermocapillary convection for an upward facing heater against buoyancy^48^,^49^,^50^. Regarding boiling performance, the Marangoni force is known to be an undervalued force, because there is a dominant buoyancy acting on nucleating bubbles in a gravitational environment^48^,^49^,^50^,^51^. However, small bubbles, of the order of a few tens of microns, can be dominated significantly by the Marangoni effect because the gravitational force is comparatively dominant for larger bubbles^52^. Thus, we speculate that the Marangoni effect can contribute to convective boiling with small nucleation sites resulting from *σ* lowering. The sequential dramatic wall superheat decrease can significantly attenuate the detrimental Marangoni force on a surface without dominant hemi-wicking effects. However, on a nano-inspired surface with hemi-wicking, the degree of Marangoni attenuation must be less dependent on *σ* lowering than on a non-wicking surface due to insensitivity to the shrinkage of nuclei (as discussed in Fig. 6) and subsequent lower variation in wall superheat, resulting in a temperature gradient along the nuclei. Thus, the improvement of HTC_*i*_/HTC_*DI*_ can be weakened when we superpose the two strategies of enhancing bulk liquid accessibility and using near-field interfacial liquid refreshing by hemi-wicking.

## Conclusions

Enhancement of boiling, a powerful and feasible heat- and energy-transferring technology, is a challenging issue for the development of higher-efficiency and next-generation energy systems. Perceiving the peculiarities of nano-inspired interfaces and their functionalities, we demonstrated the independent and synergetic impact of *interface-induced* liquid accessibility via nanostructures on boiling enhancement and examined the combined effect with *bulk* liquid accessibility controlled by a surfactant. The role of interfacial hemi-wicking, a hydrodynamic functionality inspired by nanostructures, can be a plausible clue to answering remaining questions related to CHF improvement. The CHF on nano-inspired surfaces could be well predictable as a function of the characteristics of the intrinsic morphology and corresponding wickability.

Differing from unconditional surface roughening, morphologically driven hemi-wicking can lead to dramatic CHF enhancement even with passive liquid accessibility reinforcement by lowering the surface tension of a working fluid. Moreover, the strategies of surface roughening for interfacial hemi-wicking and surface tension control for passive liquid supply were separately and simultaneously beneficial for the decrease in wall superheat. In particular, there was a unique behavior whereby comparative HTC enhancement was attenuated when we superposed *far-field* liquid feeding with interfacial liquid refreshing with superhydrophilicity due to the difference in effective cavity size reduction and its consequent impact on wall superheat variation. Manipulations of the hydrodynamics as a strategy for possible further improvement of liquid accessibility can be a promising approach for enhancement. As such, novel nano- and material technologies can be adopted to provide the required interfacial functionality, a so-called *smart interface*, for breakthroughs in boiling performance and their application in various heat- and energy-transferring systems.

## Methods

### Local temperature sensor with *in situ* nanostructures

**Figure 2:**
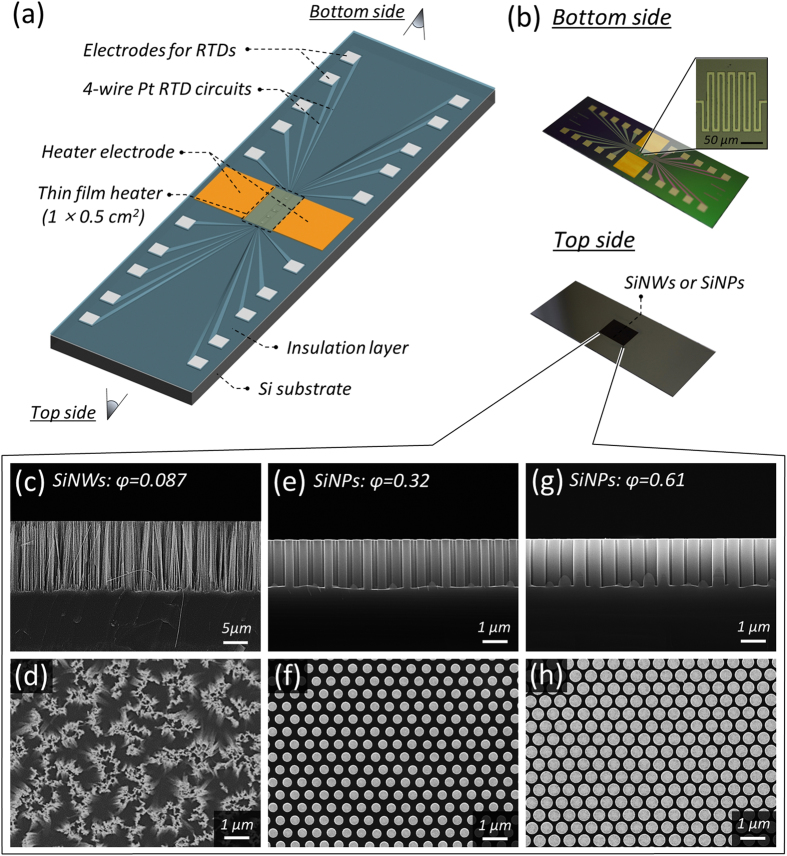
Local temperature-measuring sensor with *in situ* nanostructures. (**a**) Schematic diagram of the sensor. (**b**) Photograph of the completed sensor. (**c**,**d**) Silicon nanowires (SiNWs) with solid fraction *φ* = 0.087: average diameter *d*_*avg*_ = 100 nm, average pitch *p*_*avg*_ = 300 nm, and average height *h*_*avg*_ = 15 μm. (**e**,**f**) Silicon nanopillars (SiNPs) with *φ* = 0.32: *d*_*avg*_ = 360 nm, *p*_*avg*_ = 610 nm, and *h*_*avg*_ = 2 μm. (**g**,**h**) SiNPs with *φ* = 0.61: *d*_*avg*_ = 500 nm, *p*_*avg*_ = 610 nm, and *h*_*avg*_ = 2 μm^12^.

**Figure 3:**
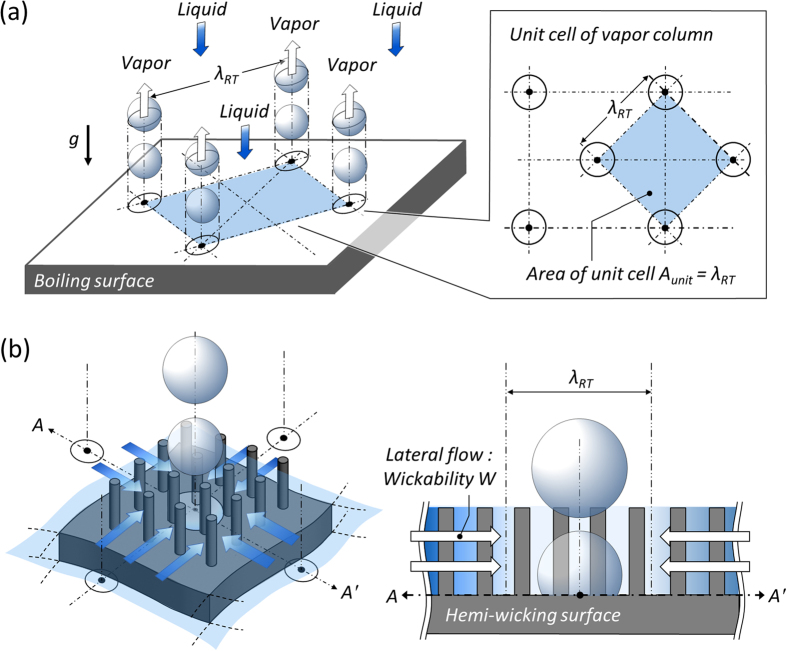
Schematic diagrams of bulk and interface-inspired hydrodynamics. (**a**) Perspective and top view of the bulk hydrodynamics showing *λ*_*RT*_-spaced array of locations of vapor rise and superpositioned liquid on an upward-facing horizontal surface^24^. This figure is redrawn with permission. Copyright 2001, Elsevier^38^. (**b**) Interface-inspired hemi-wicking and its effects on interfacial liquid refreshing towards the vaporizing region.

**Figure 4:**
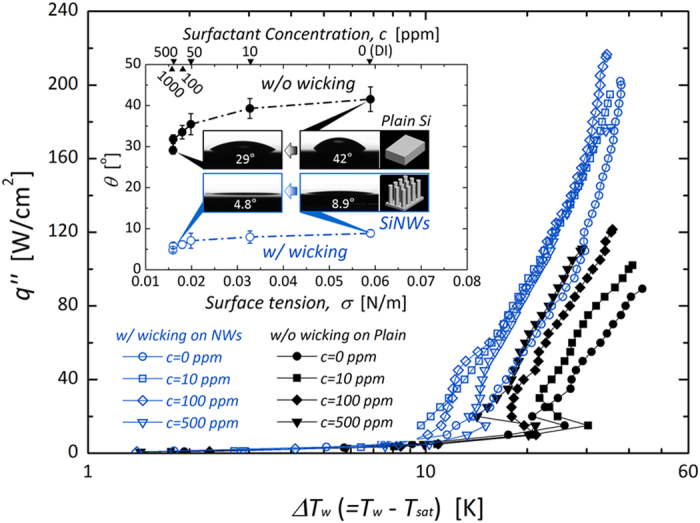
Boiling characteristics of wall superheat (*∆T*_*w*_) and applied heat flux (*q*′′) depending on *far-field* (macroscale surface tension control) and *near-field* (interfacial hemi-wicking control) liquid accessibility. For hemi-wicking conditions, vertically standing SiNWs (average diameter *d*_*avg*_ = 100 nm, pitch *p*_*avg*_ = 300 nm, and height *h*_*avg*_ = 15 μm) were used on a boiling surface. The inset presents the variation of static CAs on each surface according to the surface tension (*σ*) of DI water, controlled by the concentration of surfactant additive (*c*).

**Figure 5:**
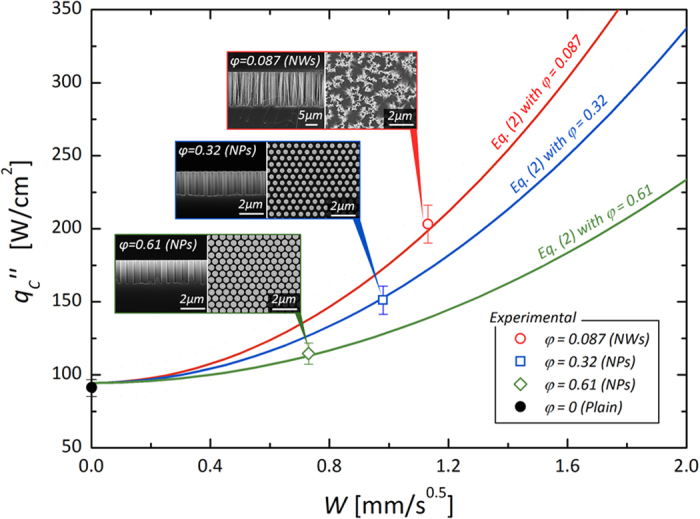
CHF estimations reflecting hydrodynamic criteria with regard to interfacial liquid refreshing based on both hydrodynamic liquid accessibilities. A parametric presentation of the solid fraction (*φ*) is demonstrated from each surface with SiNWs (*φ* = 0.087 with *d*_*avg*_ = 100 nm, *p*_*avg*_ = 300 nm, and *h*_*avg*_ = 15 μm) and SiNPs (*φ* = 0.32 with *d*_*avg*_ = 360 nm, *p*_*avg*_ = 610 nm, and *h*_*avg*_ = 2 μm and *φ* = 0.61 with *d*_*avg*_ = 500 nm, *p*_*avg*_ = 610 nm, and *h*_*avg*_ = 2 μm)^12^. Insets of scanning electron microscopy (SEM) images show cross-sectional and top views of the fabricated nanostructures.

**Figure 6:**
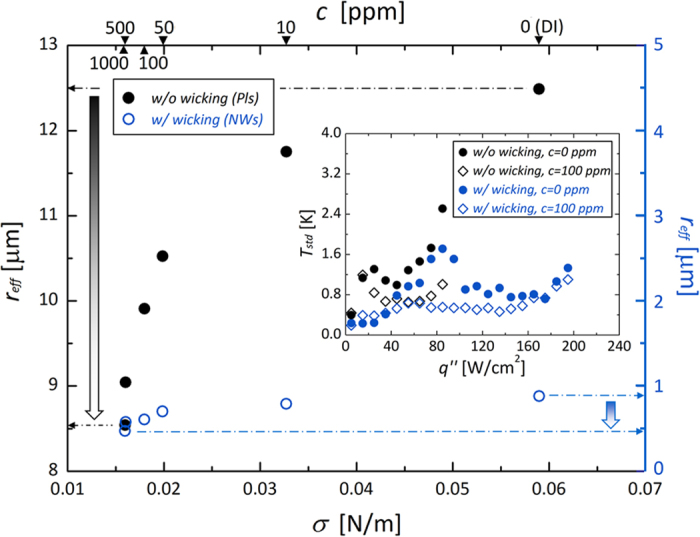
Effects of fluid surface tension on the effective cavity size. Variation of the effective cavity radius (*r*_*eff*_) in nucleate boiling on a surface without (left *y*-axis) and with (right *y*-axis) a combinational interfacial hemi-wicking effect. The inset shows the local wall temperature fluctuation. *T*_*std*_ is the standard deviation of transient wall temperature during 30 s for each steady heat flux condition.

**Figure 7:**
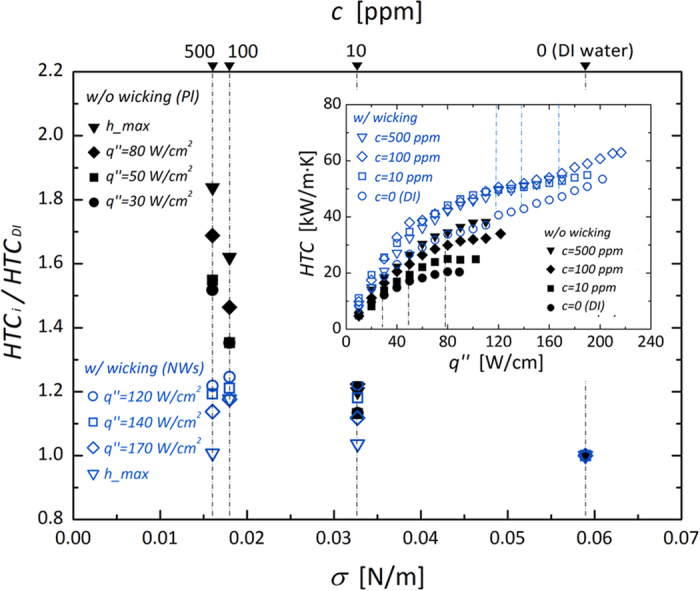
Coupling of bulk liquid feeding and near-field interfacial refreshing and their impacts on convective heat transfer in a nucleate boiling regime. (*q*′′ of 30, 50, and 80 W/cm^2^ on a surface without hemi-wicking, and 120, 140, and 170 W/cm^2^ on a surface with hemi-wicking). The inset shows overall HTCs with heat fluxes according to variation in surface tension.

**Figure 8:**
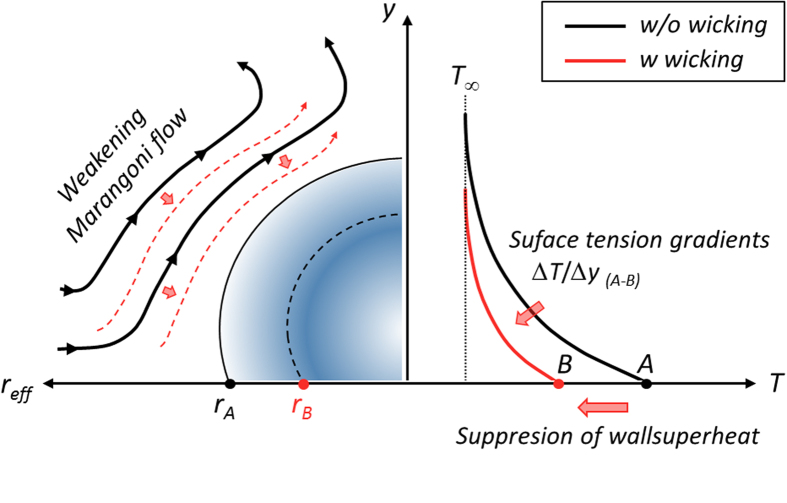
Employment of nano-interface and associated nucleate boiling heat transfer. Detrimental Marangoni flow around a nucleated bubble. Attenuation of surface tension-induced flows due to smaller nucleation and subsequently less wall superheat^56^. In schematics black and red lines indicate characteristics (flow and temperature distributions) for without and with hemi-wicking, respectively.

The devised local temperature sensor (Fig. 2(a,b)) consisted of five sets of four-wire resistance temperature detectors (RTDs), a thin film heater, and nanostructures synthesized *in situ* on the bare surface of the sensor^11^,^12^. The sensor was fabricated on a 500-μm-thick p-type silicon substrate (boron-doped, (100) orientation, 1–10 Ω·cm resistivity). The substrate was cleaned in piranha solution (3:1 mixture of H_2_SO_4_ and H_2_O_2_ by volume) for 40 min and was further sequentially cleaned with acetone and methanol for 5 min each using a sonicator. After cleaning, serpentine-shaped platinum (Pt) RTDs with a line width of 6 μm were formed by lift-off. An insulating oxide-nitride-oxide multilayer was deposited on the RTDs. After removal of the insulating layer from the RTD electrodes, an 800-nm-thick indium tin oxide (ITO) layer was deposited with sequential etching of the ITO for a 0.5 × 1.0 cm^2^ heater formation. Gold electrodes were formed on both tips of the ITO by Au lift-off. An ~1.5 Ω difference was observed for each RTD with a 1 °C change. In this study, the local wall temperature from a RTD in the center of the heating area was evaluated for boiling characterizations.

### Synthesis of nanostructures

A silicon substrate was cleaned by sequential sonication in acetone and methanol solutions, and was further cleaned in piranha solution^17^,^53^,^54^. To synthesize SiNWs, the substrate was immersed in 5 mM AgNO_3_ and 4.8 M F solution for 1 min to form a Ag^+^ coating. After rinsing the substrate with DI water, the sensor was dipped into a solution of 4.8 M HF and 0.1 M H_2_O_2_. The Si coated by reduced Ag was oxidized to SiO_2_, and the HF solution selectively dissolved the SiO_2_. The Si substrate coated by Ag was then selectively etched, and the remaining portions formed vertically aligned SiNWs. The reduced Ag on the substrate was finally removed with nitric acid. For SiNP synthesis, we used the Langmuir–Blodgett method with 610-nm-diameter polystyrene (PS) nanospheres (Invitrogen, USA) and obtained a hexagonally close-packed monolayer of PS on the air–water interface^12^. The monolayer of PS nanospheres was transferred to the target Si substrate by scooping it up^55^. PS nanospheres on the substrate were converted into non-close-packed ones by shaving their external surfaces with an O_2_ plasma, and a gold layer was deposited by E-beam evaporation. The substrate underneath the gold layer was etched in a mixture of 5 M HF and 0.5 M H_2_O_2_. After etching, the gold layer and remaining PS were removed. The characteristic lengths of SiNPs were controlled by the initial diameter of PS for the pitch of SiNPs and the diameter of the remaining PS for that of SiNPs. The heights of these structures were controlled by etching time, and the SiNWs (Fig. 2(c,d)) and SiNPs (Fig. 2(e–h)) had heights of 15 μm and 2 μm, respectively, which met the corresponding hemi-wicking criteria (see Supplementary Information).

### Morphology Characterization

Morphological characteristics of the nanostructures were evaluated by scanning electron microscopy (SEM) measurement and image processing. The measurements and data reduction were conducted using field-emission SEM (FE-SEM, JSM-7001F, JEOL, Japan).

### Evaluation of static contact angle and hemi-wicking

Static CAs were measured with a goniometer (KSV CAM-200, KSV Ins., Finland). Droplet images were collected with a high-speed camera using a frame interval of 2 ms and a resolution of 512 × 480 pixels, and static CAs were analyzed automatically with a calibrating program. Measurements were conducted using a 2.5-μL droplet of DI water, and were repeated at least seven times for averaging characteristics in each case. For the hemi-wicking evaluation, the substrate was located on a horizontal plate, and 5 μL of DI water was dropped on the substrate. The liquid propagation was recorded with a high-speed camera (M310, Dantec Dynamic, Denmark) at 100 fps. The displacement between the wicking-front line and droplet contact line was evaluated in a post-imaging process, and the consequent wicking coefficients, *W*, were evaluated by averaging eight wicking distances measured along octagonal radial lines^12^,^15^.

### Pool boiling experiments

For boiling performance evaluation, we conducted pool boiling experiments using the devised sensor with *in situ* SiNWs or SiNPs. Deionized (DI) water was used as the working fluid, and all experiments were conducted at saturation conditions under ambient pressure (*i.e.*, 100 °C of DI water at 1 atm). For manipulation of *bulk* liquid accessibility, the surface tension characteristics of the DI water were changed using a nonionic surfactant (FS-3100, DuPont, USA), which caused no significant change in physical properties except for surface tension. A detailed explanation on experimental setup and procedure is described in Supplementary Information.

## Additional Information

**How to cite this article**: Kim, B. S. *et al*. Nano-inspired fluidic interactivity for boiling heat transfer: impact and criteria. *Sci. Rep.* **6**, 34348; doi: 10.1038/srep34348 (2016).

## Supplementary Material

Supplementary Information

## Figures and Tables

**Figure 1:**
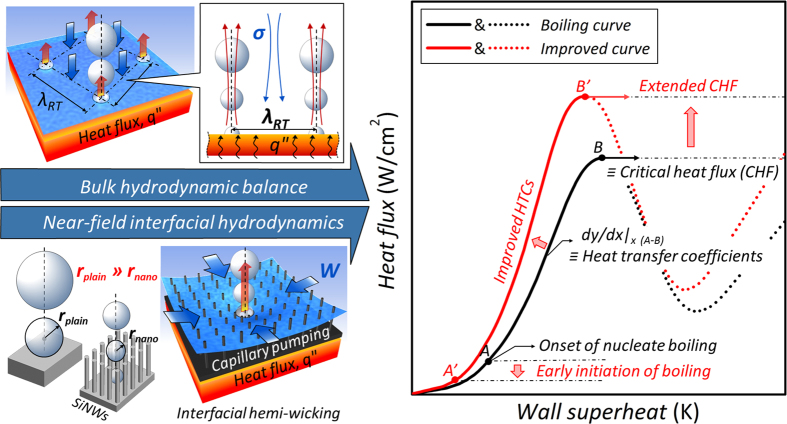
Descriptions of bulk and interface-inspired hydrodynamic behaviors in boiling heat transfer and associated boiling performance. The bulk hydrodynamic aspect is determined by liquid surface tension, *σ*, and the Rayleigh–Taylor wavelength, *λ*_*RT*_, confining the characteristic distance between upward vapor columns and counteracting downward liquids, which is related to the bulk hydrodynamic balance. Otherwise, interface-induced near-field hydrodynamics can be explained with an intuitive roughness effect and its subsequent static and dynamic wetting characteristics on an interface. Here, static and dynamic wetting can be indicated by the apparent static contact angle (CA) and morphologically induced hemi-wicking, respectively, which affect effective nucleation sizes (*r*). Boiling curves represent performance factors, including the critical heat flux (CHF), the heat transfer coefficient (HTC) and the onset of nucleate boiling^11^.
