# Supplementary material for: Nano-inspired fluidic interactivity for boiling heat transfer: impact and criteria
Source: Sci Rep. 2016 Oct 6;6:34348. doi: 10.1038/srep34348 (PMC5052621; doi:10.1038/srep34348)
Supplement: Supplementary Information [file srep34348-s1.doc]

***Supplementary Information (Scientific Reports)***

**Nano-inspired fluidic interactivity for boiling heat transfer: impact and criteria**

# Beom Seok Kim,^1^ Geehong Choi,^2^ Sangwoo Shin,^3^ Thomas Gemming,^1^ Hyung Hee Cho^2,*^

^1^*IFW Dresden, P. O. Box 270116, 01171 Dresden, Germany*

^2^*Department of Mechanical Engineering, Yonsei University, Seoul 03722, Korea*

^3^*Department of Mechanical and Aerospace Engineering, Princeton University, Princeton, New Jersey 08544, Unites States*

^*^ Corresponding author

Tel.: +82 2 2123 2828

Fax: +82 2 312 2159

E-mail: hhcho@yonsei.ac.kr

**Synthesis of nanostructures on a boiling surface**

Nanostructures were synthesized on the bare surface of the sensor after sensor fabrication. Top-down metal-assisted chemical etching (MaCE) was used to synthesize vertically aligned silicon nanowires (SiNWs) and silicon nanopillars (SiNPs)^1,2,3^. The substrate was cleaned by sequential sonication in acetone and methanol solutions, and was further cleaned in piranha solution. To synthesize SiNWs, the substrate was immersed in 5 mM AgNO_3_ and 4.8 M HF solution for 1 min to coat with Ag^+^ (AgNO_3_ was ionized to Ag^+^ and NO_3_^-^ in aqueous solution, and Ag^+^ ions were reduced on the Si substrate). After rinsing the substrate with DI water, the sensor was dipped into a solution of 4.8 M HF and 0.1 M H_2_O_2_. During the process, Ag ions act as a catalyst to generate holes through a cathode reaction (reduction of H_2_O_2_ to H_2_O). The electric potential of Si is lower than that of Ag; thus, the holes are transferred to the Si substrate. The local Si substrate coated by reduced Ag was oxidized to SiO_2_, and the HF solution selectively dissolved the SiO_2_. The Si substrate coated by Ag was then selectively etched, and the remaining portions formed vertically aligned SiNWs. The reduced Ag on the substrate was finally removed with nitric acid.

To manipulate the characteristic lengths of the nanostructures, we used nanosphere lithography combined with MaCE to synthesize SiNPs^4^. Through the Langmuir–Blodgett method with 610-nm-diameter polystyrene (PS) nanospheres (Invitrogen, USA), we first obtained a hexagonally close-packed monolayer of PS on the air–water interface. The monolayer of PS nanospheres was transferred to the Si substrate by scooping it up^5^. After drying the substrate by natural convection, PS nanospheres on the substrate were converted into non-close-packed ones by shaving their external surfaces with an O_2_ plasma while maintaining their location and arrangement. Vacant areas between the remaining PS nanospheres were then covered with gold deposited by E-beam evaporation. The substrate underneath the gold layer was etched with a mixture of 5 M HF and 0.5 M H_2_O_2_. This process allows the gold layer, as the catalytic layer in MaCE, to sink vertically into the Si substrate, analogous to the function of reduced Ag in the preparation of SiNWs. After etching, the gold layer and remaining PS were removed. The characteristic lengths of SiNPs were controlled by the initial diameter of PS for the pitch of SiNPs and the diameter of the remaining PS for that of SiNPs. During the synthesis of these nanostructures, the etching solutions used for MaCE can damage the sensor circuits. Thus, we used a Teflon cover with O-rings with a selective open area on the rear of the sensor substrate.

**Roughness factor and solid fraction of nanostructure-employed surfaces**

The roughness factor *r* and the solid fraction *φ* on surfaces indicate the ratio of the actual surface area to the projected one and the area fraction remaining dry below a liquid droplet. The vertically aligned SiNWs and SiNPs can be assumed to be a forest filled with densely distributed circular pillars; each structure is shown schematically in Fig. S1(a) and (b), respectively. Here, *φ* can be described as the top area of the circular pillar to the entire in a unit area. According to the schemes, *r* and *φ* dry can be expressed as follows:

| SiNWs (Fig. S1(a)): 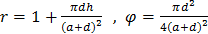 | (S1) |
| --- | --- |
| SiNPs (Fig. S1(b)): 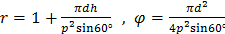 | (S2) |

where *d*, *h*, *a*, and *p* are the diameter of a pillar, height of a pillar, the distance between pillars, and their pitch, respectively. Dimensional values of the characteristic lengths of the synthesized structures were obtained from field-emission scanning electron microscopy (FE-SEM) measurements.


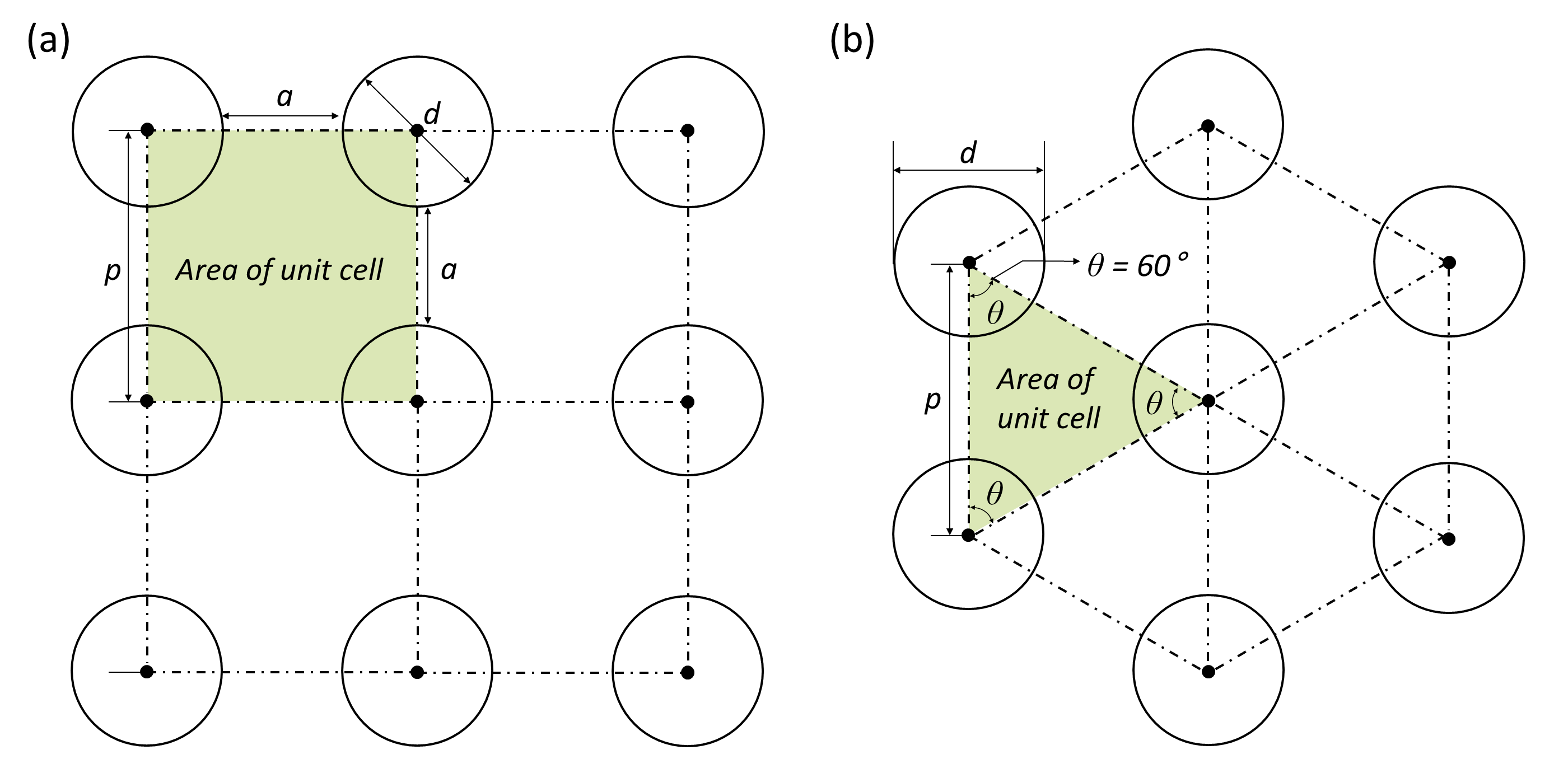


**Figure S1.** Schematic diagrams describing vertically aligned silicon nanowires (SiNWs) and regularly arranged silicon nanopillars (SiNPs).

**Prerequisite for hemi-wicking**

Morphologically induced hemi-wicking has proven to be a powerful mechanism to induce strong momentum of liquid flow on surfaces, and using these nano-inspired structures is a practical method to meet the critical prerequisite for hemi-wicking as *θ_c_* > *θ^*^*, where cos*θ_c_* = (1-*φ*)/(*r*-*φ*)^6,7^. Here, *θ_c_*, *θ^*^*, *φ*, and *r* are the critical contact angle (CA) needed as a prerequisite for wicking initiation, equilibrium CA on an ideal surface, solid fraction remaining dry when a solid–liquid interface contacts a liquid droplet, and roughness factor as the ratio of actual to projected surface area, respectively^4,6,7,8^. To meet the criteria specifications, we manipulated the heights of the nanostructures. The heights of these structures were controlled by etching time; the SiNWs and SiNPs used had heights of 15 μm and 2 μm, respectively.

**Evaluation of wickability, *W***^4^

For the quantitative evaluation of hemi-wicking (*i.e.*, wickability, *W*), we can consider a fundamental model suggested by Washburn as follows^9,10^:

| 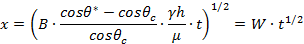 | (S3) |
| --- | --- |

where *x*, *B*, *θ^*^*, *θ_c_*, *γ*, *h*, *μ*, *t*, and *W* are the wicking distance, empirically determined constant, equilibrium contact angle on an ideal plain surface, critical contact angle (cos*θ_c_* = (1-*φ*)/(*r*-*φ*) where *φ* and *r* indicate roughness factor defined as the ratio of the actual to projected surface area and the solid fraction of the solid-liquid interface contacting the liquid droplet, respectively), surface tension of the liquid, characteristic length of interfacial structures (wicking channels), viscosity of the liquid, time, and wickability, respectively. Based on this relation, wicking propagation distance (*x*) was computed on each manipulated nano-inspired surface.

**
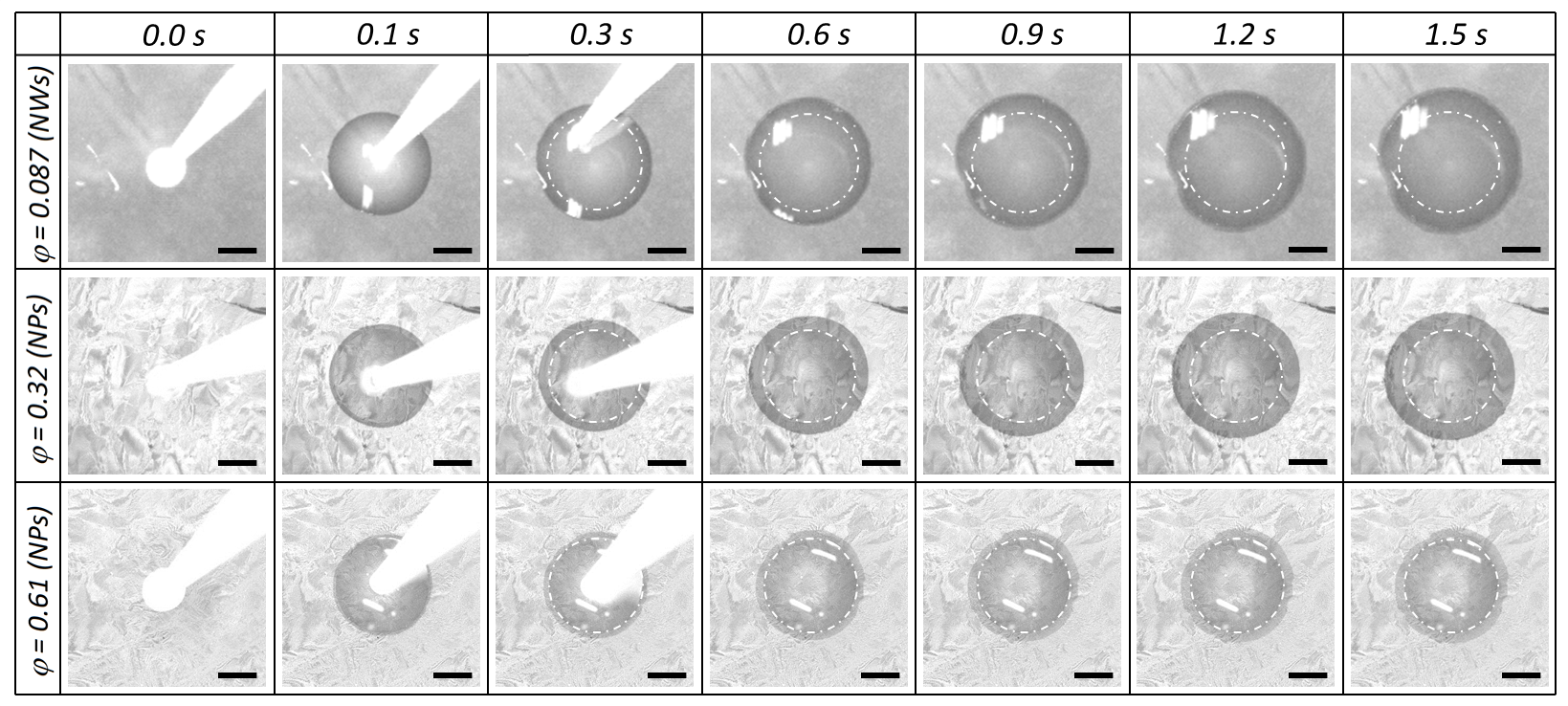
**

**Figure S2.** Hemi-wicking on the manipulated nano-inspired surfaces. White dot lines indicate the droplet edge. Scale bars indicated in each image are 2 mm^4^.


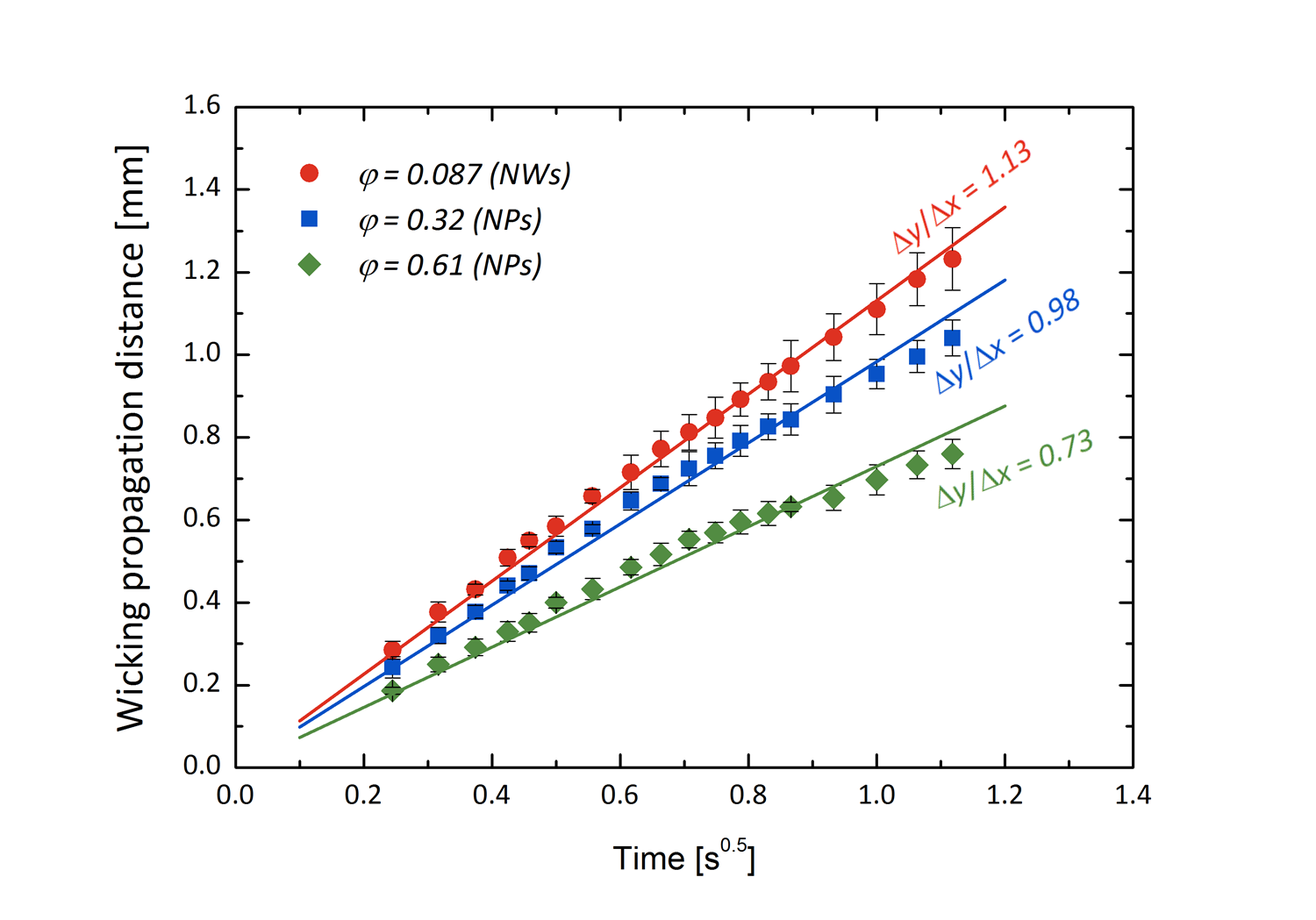


**Figure S3.** Hemi-wicking propagation plots. The wickability (or wicking coefficient) *W* is defined by the average slope of each linear fitting curve of wicking distance versus the square root of time^4^.

The propagation phenomena of DI water on the manipulated surfaces were monitored with a high-speed camera (M310, Dantec Dynamic, Denmark) at 100 fps. Then, quantitative data of the propagation distance were extracted from the recorded photo-images. During measurements, a substrate was located on a horizontal plate, and 5 μL of DI water was dropped on the center of the substrate. The displacement between the wicking-front line and droplet contact line was evaluated in a post-imaging process, and the propagating distances were evaluated by averaging eight wicking distances measured along octagonal radial lines^4,11^. Figures S2 and S3 show images of liquid propagation with time after the dropping of a droplet (DI-water) and propagation distances evaluated on surfaces. From the graphs of the distance versus time (s^0.5^), the wickability can be indicated by the degree of inclination of each linear fitting curve. For the hemi-wicking and CHF model described in Eq. (2), the obtained wickability values of 1.12 mm/s^0.5^, 0.98 mm/s^0.5^, and 0.73 mm/s^0.5^ were used for corresponding surface conditions of SiNWs (*φ* = 0.087), SiNPs (*φ* = 0.32), and SiNPs (*φ* = 0.61), respectively.

**Pool boiling facility and experiments**^4,8^

Figure S4 describes the pool boiling experimental facility. The pool boiling chamber was made of stainless steel, and a test section was made of Teflon with low thermal conductivity (0.23 W/m·K) to minimize heat conduction loss. Spring probes and metallic connectors were installed in the test section to acquire RTD signals and to apply an electric current, respectively. The portion of the test section just below the installed

**
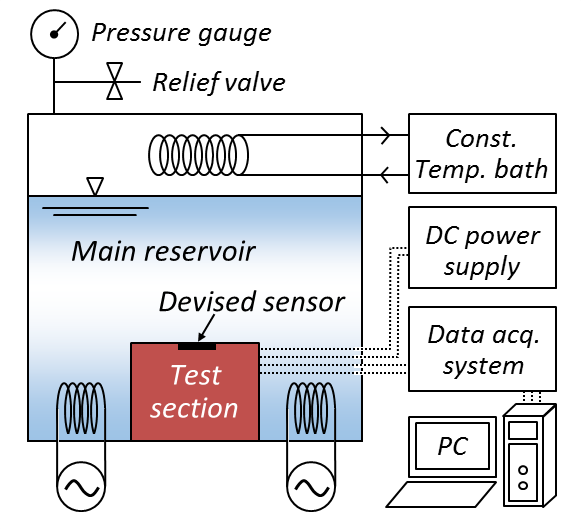
**

**Figure S4.** Schematic diagram of the experimental apparatus for the evaluation of boiling heat transfer with a test section for installing the devised local temperature measuring sensor.

sensor and a cover plate were made of macerite ceramic SP with a thermal conductivity of 1.6 W/m·K and a high melting point, over 1000°C, to prevent conductive heat loss and breakdown as the test conditions approached the CHF limitation, respectively. The test section was completely sealed using room-temperature vulcanization silicone (RTV Ultra Blue – 77BR, Permatex, USA). A direct current (DC) power supply (200 V, 10 A) was used to supply a heat flux by adjusting the current. DI water was the working fluid and it was controlled under a saturated condition (100 °C) at atmospheric pressure (1 atm) in the pool boiling reservoir. Gases dissolved in the reserved DI water were removed before boiling experiments. Using the devised sensor, we gradually increased the heat flux from 0 to CHF conditions by a step-wise increase in DC electric current that passed through the ITO heater. When steady conditions at a controlled heat flux were confirmed, RTD signals were recorded by a module (SCXI 1503 and DAQ 6259, National Instrument, USA) for 30 s with a frequency of 1000 Hz and were averaged. Other electric signals were monitored by a data acquisition module (34970A, Agilent Technology, USA) for the same period at a frequency of 2 Hz.

**Wicking-CHF model**

The wicking-CHF model formulated in Eq. (2) could be modeled quantitatively by considering the refreshing rate of liquid-phase working fluids towards a solid–liquid interface (*i.e.*, boiling surface with *in* *situ* nanostructures). Here, the driving force for the refreshing of a liquid is the morphologically induced hemi-wicking. For analytical demonstrations of hemi-wicking, we discussed above a morphological prerequisite of the interface to provide hemi-wicking. With the prerequisite of the employed interfacial structures, we can then reach a certain criterion that the maximum capacity of heat dissipation should be balanced with the thermodynamically absorbed heat during a phase change of the refreshing liquids on the boiling surface. We can calculate the volumetric capacity of the porous interface considering the solid fraction of *φ*. This was already defined as the solid fraction of the solid–liquid interface contacting the liquid. Thus, (1−*φ*)·*h* identifies the volumetric capacity with the height of manipulated nanostructures on a confined unit area. Because *W*, the wickability, indicates the ability of liquid propagation by hemi-wicking^9,10,12^, it is reasonable to approximate the refreshing rate of a working fluid (liquid-phase) by *W*^2^ (expansion rate of wetted area by liquid propagation per unit second) in the porous volume of the interface layer. Here, the interface layer should be confined to the solid−liquid interface with a thickness of *h*, which is the characteristic height of the nanopillar structures. The thermodynamic equilibrium of CHF can then be explained considering the hydrodynamic refreshing of a liquid towards the interface. When a liquid perfectly propagates by wicking through the interface, the total amount of the refreshing liquid can be expressed simply as (1−*φ*)·*h·W^2^*. Then, the total heat dissipation capacity of the confined unit area (*i.e.*, the amount of heat absorption for a phase change of the liquid confined in the area) can be expressed as (1−*φ*)·*h·W^2^×*(*l·ρ_l_*), where *l* and *ρ_l_* are the latent heat of vaporization and density of the working fluid, respectively. In this, we assume that hemi-wicking occurs with a uniform velocity profile along a vertical direction from 0 (at the substrate) to *h* (top of wicking-inducing structure) within the interface layer, and the refreshing of a liquid-phase working fluid is even in a unit area. We confirm that the empirical constant of *C_1_* was valid, with the variation of the solid fraction, as *C_1_*·*h* = 0.1021 in the range of *h*, as a characteristic length of a wicking channel, from 2 micron to 15 micron^4^.

Taking into account the unit area of the approach, it should be confined by considering hydrodynamic instability. On a confined boiling surface, the hydrodynamic stability should be guaranteed by balancing up-flowing vapor columns and down-flowing liquid^13,14^. According to the hydrodynamic instability concept, CHF occurs when the hydrodynamic stability becomes unstable, causing a large retarding force on down-flowing liquid^15^. Therefore, the wicking-CHF model can be confined considering the hydrodynamic stability by accepting the Rayleigh−Taylor interfacial stability wavelength as the unit length-scale for the smallest cell distance. Figure 3(a) describes the physical definition of the characteristic wavelength of the two counteracting fluids.

**Experimental determination of critical heat flux (CHF)**^4^

As an applied heat flux increases, numerous and vigorous bubble generation can be detected. When the excessive and fast bubble generations proceed, a vapor layer forms on a boiling surface due to their merging and prevents re-wetting of the surface. This results in abrupt increase of wall temperature accompanying fierce fluctuations since the vapor layer prevents the heat flux from dissipating towards the outward environment^16^. The significant temperature fluctuation can be clearly monitored just before a critical condition of CHF^17^. When heat flux approaches CHF, local wall temperatures fluctuate vigorously. In this study, we stopped increasing heat flux when the temperature fluctuation larger than 15 K could be monitored. CHF was quantitatively evaluated by adding the heat flux measured at a steady state condition just before the occurrence of the significant temperature variation (more than 15 K) and half an increment of heat flux from the previous step, just before increasing the heat flux^4,8,18^.

**Uncertainty analysis**^4^

The uncertainty analysis was performed for the principal variables described in the manuscript as well as the fundamental dimensional estimations. All of errors were estimated with a confidence level of 95% with amounts of measured data set for each variable. Errors in the dimensional estimation of the fabricated sensor and temperature measurement by conventional K-type thermocouples that we used were 0.2% and 1.32 K, respectively. A principal error source in boiling experiment results from heat loss pertaining to systematic installation of the test sections and intrinsic conductive heat loss. Because it is not easy to directly estimate heat loss, we indirectly estimated the heat loss by a computational analysis using boundary conditions obtained from experiments, and then took it into the estimation of uncertainty of applied heat flux. The heat loss was calculated using 3-dimensional numerical analysis using commercial CFD code (Fluent v.6.3.26, ANSYS). We compared the numeric results with experimental ones especially on local temperature from the center point of heating area. Iterative calculations were conducted until the deviation between the analytical and experimental results came to within 1.0%. The uncertainties on principal variables were evaluated based on the relationships used to derive for heat flux and local wall temperature as follows^19^:

| 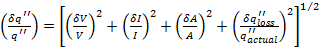 | (S4) |
| --- | --- |
| 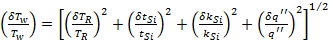 | (S5) |
| 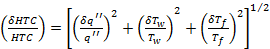 | (S6) |

where *V*, *I*, *A*,
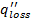
,
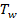
,
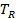
,
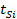
,
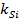
, *HTC* and *T_f_* are the voltage drop from the thin film heater, applied currents, heating area, heat loss estimated by the analysis, wall temperature, temperature measured from RTD sensor, thickness of Si substrate, thermal conductivity of Si, heat transfer coefficient, and temperature of liquid phase fluid, respectively. From the procedures, we confirm that the estimated uncertainties of heat flux, local wall temperature, and heat transfer coefficient are 6.4%, 6.8% and 7.2%, respectively^4,8^.

Wicking coefficient was calculated through the post analyses using a propagation height image, which was calibrated with a pixel resolution of 0.1 × 0.1 mm^2^/pixel. This image processing method has inherent uncertainties resulting from pixel resolution of images and time-step errors during the recording, which correspond to respective spatial and temporal uncertainty based on the camera’s specification. According to the definition of the wicking coefficient (Eq. S3)), we can analyze the uncertainty as a function of related physical factors as follows^10,12^:

| 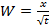 from Eq. (S3), 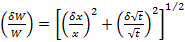 | (S7) |
| --- | --- |

where *W*, *x*, and *t* mean the wicking coefficient, wicking distance, and time, respectively. We took each error considering allowable maximum errors pertaining to image resolution and to exposure time as the spatial uncertainty and temporal uncertainty, respectively, in Eq. (S7). Each error was 3.47% and 3.11% according to the image taken at 4 s, and thus the uncertainty of wicking coefficient was 4.60% ^4^.

**References**

1. Huang, Z., Geyer, N., Werner, P., de Boor, J. & Gösele, U. Metal-assisted chemical etching of silicon: A review. *Adv. Mater.* **23,** 285-308 (2011).

2. Kim, B. S., Shin, S., Shin, S. J., Kim, K. M. & Cho, H. H. Micro-nano hybrid structures with manipulated wettability using a two-step silicon etching on a large area. *Nanoscale Res. Lett.* **6,** 333 (2011).

3. Kim, S. & Khang, D. Bulk micromachining of Si by metal-assisted chemical etching. *Small* **10,** 3761-3766 (2014).

4. Kim, B. S., Lee, H., Shin, S., Choi, G. & Cho, H. H. Interfacial wicking dynamics and its impact on critical heat flux of boiling heat transfer. *Appl. Phys. Lett.* **105,** 191601 (2014).

5. Oh, J. R., Moon, J. H., Yoon, S., Park, C. R. & Do, Y. R. Fabrication of wafer-scale polystyrene photonic crystal multilayers via the layer-by-layer scooping transfer technique. *J. Mater. Chem.* **21,** 14167-14172 (2011).

6. Bico, J., Tordeux, C. & Quéré, D. Rough wetting. *EPL* **55,** 214-220 (2001).

7. Bico, J., Thiele, U. & Quéré, D. Wetting of textured surfaces. *Colloid Surf. A-Physicochem. Eng. Asp.* **206,** 41-46 (2002).

8. Kim, B. S. *et al.* Stable and uniform heat dissipation by nucleate-catalytic nanowires for boiling heat transfer. *Int. J. Heat Mass Transf.* **70,** 23-32 (2014).

9. Washburn, E. W. The dynamics of capillary flow. *Phys. Rev.* **17,** 273-283 (1921).

10. Tas, N. R., Haneveld, J., Jansen, H. V., Elwenspoek, M. & van den Berg, A. Capillary filling speed of water in nanochannels. *Appl. Phys. Lett.* **85,** 3274-3276 (2004).

11. Kim, B. S., Shin, S., Shin, S. J., Kim, K. M. & Cho, H. H. Control of superhydrophilicity/superhydrophobicity using silicon nanowires via electroless etching method and fluorine carbon coatings. *Langmuir* **27,** 10148-10156 (2011).

12. Ishino, C., Reyssat, M., Reyssat, E., Okumura, K. & Quéré D. Wicking within forests of micropillars. *EPL* **79,** 56005 (2007).

13. Lienhard, J. H. & Dhir, V. K. *Extended hydrodynamic theory of the peak and minimum pool boiling heat fluxes* (NASA Report No. CR2270, 1973).

14. Zuber, N. Y. *Hydrodynamic aspects of boiling heat transfer* (AEC Report AECU-4439 No. 0225960, 1959).

15. Lu, M. C., Chen, R. K., Srinivasan, V., Carey, V. & Majumdar, A. Critical heat flux of pool boiling on Si nanowire array-coated surfaces. *Int. J. Heat Mass Transf.* **54,** 5359-5367 (2011).

16. Goldstein, R. J. *et al.* Heat transfer - A review of 2003 literature. *Int. J. Heat Mass Transf.* **49,** 451-534 (2006).

17. Haramura, Y. & Katto, Y. A new hydrodynamic model of critical heat flux, applicable widely to both pool and forced convection boiling on submerged bodies in saturated liquids. *Int. J. Heat Mass Transf.* **26,** 389-399 (1983).

18. Rainey, K. N. & You, S. M. Effects of heater size and orientation on pool boiling heat transfer from microporous coated surfaces. *Int. J. Heat Mass Transf.* **44,** 2589-2599 (2001).

19. Kline, S. J. The purposes of uncertainty analysis. *J. Fluids Eng.* **107,** 153-160 (1985).
